# Detection of clade 2.3.4.4b highly pathogenic H5N1 influenza virus in New York City

**DOI:** 10.1128/jvi.00626-24

**Published:** 2024-05-15

**Authors:** Philip S. Meade, Pooja Bandawane, Kaitlyn Bushfield, Irene Hoxie, Karla R. Azcona, Daneidy Burgos, Sadia Choudhury, Adama Diaby, Mariama Diallo, Kailani Gaynor, Aaron Huang, Kadiatou Kante, Shehryar N. Khan, William Kim, Paul Kehinde Ajayi, Ericka Roubidoux, Sasha Nelson, Rita McMahon, Randy A. Albrecht, Florian Krammer, Christine Marizzi

**Affiliations:** 1 Department of Microbiology, Icahn School of Medicine at Mount Sinai, New York, New York, USA; 2 Center for Vaccine Research and Pandemic Preparedness (C-VaRPP), Icahn School of Medicine at Mount Sinai, New York, New York, USA; 3 New York City Virus Hunters Program, BioBus, New York, New York, USA; 4 Department of Host Microbe Interactions, St. Jude Children’s Research Hospital, Memphis, Tennessee, USA; 5 Animal Care Centers of New York, New York, New York, USA; 6 Wild Bird Fund, New York, New York, USA; 7 The Global Health and Emerging Pathogens Institute, Icahn School of Medicine at Mount Sinai, New York, New York, USA; 8 Department of Pathology, Molecular and Cell Based Medicine, Icahn School of Medicine at Mount Sinai, New York, New York, USA; 9 Ignaz Semmelweis Institute, Interuniversity Institute for Infection Research, Medical University of Vienna, Vienna, Austria; St. Jude Children's Research Hospital, Memphis, Tennessee, USA

**Keywords:** influenza, H5N1, community science, science outreach, pandemic preparedness, citizen science, participatory research, avian influenza viruses

## Abstract

Highly pathogenic avian influenza viruses of the H5N1 clade 2.3.4.4b were detected in North America in the winter of 2021/2022. These viruses have spread across the Americas, causing morbidity and mortality in both wild and domestic birds as well as some mammalian species, including cattle. Many surveillance programs for wildlife as well as commercial poultry operations have detected these viruses. In this study, we conducted surveillance of avian species in the urban environment in New York City. We detected highly pathogenic H5N1 viruses in six samples from four different bird species and performed whole-genome sequencing. Sequencing analysis showed the presence of multiple different genotypes. Our work highlights that the interface between animals and humans that may give rise to zoonotic infections or even pandemics is not limited to rural environments and commercial poultry operations but extends into the heart of our urban centers.

**IMPORTANCE:**

While surveillance programs for avian influenza viruses are often focused on migratory routes and their associated stop-over locations or commercial poultry operations, many bird species—including migratory birds—frequent or live in urban green spaces and wetlands. This brings them into contact with a highly dense population of humans and pets, providing an extensive urban animal–human interface in which the general public may have little awareness of circulating infectious diseases. This study focuses on virus surveillance of this interface, combined with culturally responsive science education and community outreach.

## INTRODUCTION

Zoonotic infections caused by highly pathogenic avian influenza (HPAI) viruses of the H5N1 subtype were first detected in Hong Kong in 1997 (1, 2). After a hiatus, human infections with these A/goose/Guangdong/1/96-like viruses were detected again in 2003 (3). Their range was initially restricted to birds in Southeast Asia, but they spread westward into the Middle East (4, 5), Europe (6–8), and Africa (8, 9) via migratory birds. In addition, these H5N1 viruses also diversified and split into many different lineages. Between 2010 and 2011, clade 2.3.4.4 viruses emerged in China and started to reassort with other avian influenza viruses, producing H5NX genotypes, of which many seemed to be of lower pathogenicity (10). These viruses were introduced to the United States (US) in 2014 and caused widespread issues in the poultry industry (10). However, in 2015, they disappeared from circulation in North America (11). A subclade of clade 2.3.4.4, namely, clade 2.3.4.4b, spread in Eurasia and Africa in 2020, this time again with an N1 neuraminidase (NA) (12), and emerged in North America via migratory birds in the winter of 2021/2022 (13–15). The clade 2.3.4.4b viruses have now spread across the Americas and have heavily impacted wild bird populations and have affected the poultry industry (16–19). In addition, infections in mammals—often leading to neurological symptoms and fatal outcomes—have been reported. This includes predatory animals and scavengers feeding on sick or dead birds (20–22). These are mostly seen as dead-end hosts. Marine mammals have also been affected, especially in South America, and mammal-to-mammal transmission is suspected in some of these outbreaks (23–25). Furthermore, clade 2.3.4.4b H5N1 seems to have caused outbreaks in fur farms in Europe in mink and foxes with potential mammal-to-mammal transmission (26–28), and recently reported cases in dairy cattle are also raising concerns. Human cases caused by clade 2.3.4.4b H5N1 so far have been rare, and only two severe infections are known in the Americas (with a low number of additional ones in Asia), which is remarkable given the extent of the spread of this virus and the potential exposure to humans (29–31).

Nevertheless, it is very important to track the spread of this virus to determine potential risk to humans. There is a need for viral surveillance in urban areas that often have plenty of green spaces and wetlands for both resident and migratory birds. This, in combination with high human population densities, creates an extensive urban animal–human interface. In this interface, pets can also be impacted, as shown by infections with H5N1 in cats and dogs (32–35). Communicating this risk to urban populations is critical. Here, we set out to detect HPAI H5N1 viruses in New York City using surveillance in wildlife rehabilitation centers as well as sampling bird feces from the environment. Our approach is based on a collaboration between research institutions, science outreach organizations, wildlife welfare nonprofit organizations, and community scientists. Community scientists working with our research team have previously reported the first detection of avian paramyxovirus 1 in New York City’s pigeon population (36). The growing interest in biodiversity protection and citizen science has resulted in initiatives that collect a massive quantity of data about birds (37, 38). However, these approaches are frequently limited to participatory data collection (38, 39). In the collaborative New York City Virus Hunters initiative described in this study, we aim to engage the community in every step of the research process. Mentored research for high school students who self-identify as members of racial or ethnic minoritized groups in science is a core element. The students work alongside expert mentors and actively engage in overall study design before safely participating in sample collection, processing, data analysis, dissemination of results, and community outreach. The outputs of this program benefit all participants (40).

## RESULTS

### Surveillance strategy and virus detection

Our sampling strategy prioritized samples collected from birds known to contract HPAI H5N1—principally wild aquatic avian species of Anseriformes (ducks, geese, and swans), Charadriiformes (gulls, terns, auks and other shorebirds) and raptors such as Accipitriformes (hawks, ospreys, and other birds of prey) and Falconidae (falcons and kestrels). Samples for this study were collected from January 2022 to November 2023. In total, 1,927 samples were collected and processed for this study. We used two sampling streams. First, 125 environmental fecal samples were collected from New York City parks and green spaces using proper personal protective equipment (masks and gloves). In addition, professional animal rehabilitators at the Wild Bird Fund (WBF) and veterinarians of the Animal Care Centers (ACC) of New York City provided four water samples (3 mL each) and 1,798 cloacal (CS), oropharyngeal (OS), and fecal swabs from urban wild and domestic birds submitted to them. From these 1,798 samples (237 fecal samples, 783 CS and 764 OS samples, and 14 samples where CS or OP was nonspecified; from 895 birds), six were found positive for HPAI H5N1. While for environmental fecal samples collected in urban parks and green spaces, the avian species is hard to determine by the appearance of the sample, CS, OS, and fecal samples provided by wildlife rehabilitation centers were documented to be from 80 different species (see Table 1). The majority were from gulls and terns (348 samples/19.35%), chicken (306 samples/17.01%), geese (247 samples/14.29%), ducks (133 samples/7.39%), hawks (112 samples/6.22%), crows and ravens (85 samples/4.72%), falcons and kestrels (53 samples/2.94%), and cormorants (43 samples/2.39%). Eighty-nine samples (4.94%) were from nonspecified species. The remaining samples and the species they were collected from are also listed in Table 1. RNA was extracted from 1,927 samples, and reverse transcription was used to generate cDNA. We then screened the cDNA preparations via a multiplex PCR using primers for the matrix (M) genomic segment, the nucleoprotein (NP) genomic segment, and for the hemagglutinin (HA) genomic segment. Primers for the HA segment were H5 HA-specific, while M and NP primers were designed to detect all known influenza A viruses. Gene products were sent for Sanger sequencing. If Sanger sequencing indicated the presence of influenza A virus, whole-genome sequencing was performed. Samples from six birds were found to be positive for HPAI H5N1, and whole genomes could be obtained. No environmental fecal samples were found positive for HPAI H5N1. No other avian influenza viruses were detected.

**TABLE 1:** Details on samples for virological analysis collected from different avian species present at Wild Bird Fund or Animal Care Centers of New York City

	Host species	Scientific name	Order taxa	Family taxa	Total samples	HPAI H5N1-positive samples
1	American bittern	*Botaurus lentiginosus*	Pelecaniformes	Ardeidae	2	
2	Cooper’s hawk	*Accipiter cooperii*	Accipitriformes	Accipitridae	22	
3	Hawk	*Accipitridae sp*.	Accipitriformes	Accipitridae	2	
4	Red-shouldered hawk	*Buteo lineatus*	Accipitriformes	Accipitridae	4	
5	Red-tailed hawk	*Buteo jamaicensis*	Accipitriformes	Accipitridae	81	1
6	Sharp-shinned hawk	*Accipiter striatus*	Accipitriformes	Accipitridae	3	
7	Dovekie	*Alle alle*	Charadriiformes	Alcidae	2	
8	American black duck	*Anas rubripes*	Anseriformes	Anatidae	2	
9	Black scoter	*Melanitta americana*	Anseriformes	Anatidae	2	
10	Brant goose	*Branta bernicla*	Anseriformes	Anatidae	12	
11	Canada goose	*Branta canadensis*	Anseriformes	Anatidae	222	3
12	Domestic duck	*Anas p. domesticus*	Anseriformes	Anatidae	8	
13	Domestic goose	*Anas a. domesticus*	Anseriformes	Anatidae	2	
14	Duck	*Anas sp*.	Anseriformes	Anatidae	98	
15	Goose	*Anatidae sp*.	Anseriformes	Anatidae	10	
16	Mallard	*Anas platyrhynchos*	Anseriformes	Anatidae	92	
17	Muscovy duck	*Cairina moschata*	Anseriformes	Anatidae	3	
18	Mute swan	*Cygnus olor*	Anseriformes	Anatidae	43	
19	Northern shoveler	*Spatula clypeata*	Anseriformes	Anatidae	3	
20	Ruddy duck	*Oxyura jamaicensis*	Anseriformes	Anatidae	9	
21	Snow goose	*Anser caerulescens*	Anseriformes	Anatidae	11	
22	Swan	*Cygnus sp*.	Anseriformes	Anatidae	5	
23	Wood duck	*Aix sponsa*	Anseriformes	Anatidae	13	
24	Great blue heron	*Ardea herodias*	Pelecaniformes	Ardeidae	6	
25	Green heron	*Butorides virescens*	Pelecaniformes	Ardeidae	1	
26	Least bittern	*Ixobrychus exilis*	Pelecaniformes	Ardeidae	2	
27	Night heron	*Ardeidae sp*.	Pelecaniformes	Ardeidae	4	
28	Yellow-crowned night heron	*Nyctanassa violacea*	Pelecaniformes	Ardeidae	8	
29	Homing pigeon	*Columba livia domestica*	Columbiformes	Columbidae	1	
30	Mourning dove	*Zenaida macroura*	Columbiformes	Columbidae	7	
31	Rock pigeon	*Columba livia*	Columbiformes	Columbidae	41	
32	American crow	*Corvus brachyrhynchos*	Passeriformes	Corvidae	51	
33	Common raven	*Corvus corax*	Passeriformes	Corvidae	8	
34	Crow	*Corvus sp*.	Passeriformes	Corvidae	2	
35	Fish crow	*Corvus ossifragus*	Passeriformes	Corvidae	18	
36	Raven	*Corvus sp*.	Passeriformes	Corvidae	6	
37	American kestrel	*Falco sparverius*	Falconiformes	Falconidae	39	
38	Kestrel	*Falco sp*.	Falconiformes	Falconidae	2	
39	Peregrine falcon	*Falco peregrinus*	Falconiformes	Falconidae	12	1
40	Loon	*Gavia sp*.	Gaviiformes	Gaviidae	2	
41	Red-throated loon	*Gavia stellata*	Gaviiformes	Gaviidae	5	
42	Arctic tern	*Sterna paradisaea*	Charadriiformes	Laridae	2	
43	Great black-backed gull	*Larus marinus*	Charadriiformes	Laridae	47	
44	Gull	*Laridae sp*.	Charadriiformes	Laridae	2	
45	Herring gull	*Larus argentatus*	Charadriiformes	Laridae	180	
46	Laughing gull	*Leucophaeus atricilla*	Charadriiformes	Laridae	52	
47	Ring-billed gull	*Larus delawarensis*	Charadriiformes	Laridae	62	
48	Seagull	*Laridae sp*	Charadriiformes	Laridae	3	
49	Guineafowl	*Numida sp*.	Galliformes	Numididae	2	
50	Osprey	*Pandion haliaetus*	Accipitriformes	Pandionidae	5	
51	Tufted titmouse	*Baeolophus bicolo*	Passeriformes	Paridae	1	
52	Northern waterthrush	*Parkesia noveboracensis*	Passeriformes	Parulidae	1	
53	White-throated sparrow	*Zonotrichia albicollis*	Passeriformes	Passerellidae	2	
54	House sparrow	*Passer domesticus*	Passeriformes	Passeridae	6	
55	Cormorant	*Phalacrocorax sp*.	Phalacrocorax	Phalacrocoracidae	10	
56	Double-crested cormorant	*Nannopterum auritum*	Suliformes	Phalacrocoracidae	33	
57	Barred rock chicken	*Gallus gallus domesticus*	Galliformes	Phasianidae	4	
58	Chicken	*Gallus gallus domesticus*	Galliformes	Phasianidae	302	1
59	Fowl	*Gallus sp*.	Galliformes	Phasianidae	36	
60	Japanese quail	*Coturnix japonica*	Galliformes	Phasianidae	3	
61	Patridge	*Arborophila sp*.	Galliformes	Phasianidae	12	
62	Pheasant	*Phasianus colchicus*	Galliformes	Phasianidae	5	
63	Quail	*Coturnix coturnix*	Galliformes	Phasianidae	11	
64	Turkey	*Phasianidae sp*.	Galliformes	Phasianidae	4	
65	Wild turkey	*Meleagris gallopavo*	Galliformes	Phasianidae	7	
66	Yellow-bellied sapsucker	*Sphyrapicus varius*	Piciformes	Picidae	4	
67	Grebe	*Podicieps sp*.	Podicipediformes	Podicipedidae	3	
68	Cory’s shearwater	*Calonectris borealis*	Procellariiformes	Procellariidae	1	
69	Great shearwater	*Ardenna gravis*	Procellariiformes	Procellariidae	2	
70	Parrot	*Psittacidae sp*.	Psittaciformes	Psittacidae	3	
71	American coot	*Fulica americana*	Gruiformes	Rallidae	5	
72	American woodcock	*Scolopax minor*	Charadriiformes	Scolopacidae	2	
73	Barred owl	*Strix varia*	Strigiformes	Strigidae	3	
74	Great horned owl	*Bubo virginianus*	Strigiformes	Strigidae	6	
75	Northern saw-whet owl	*Aegolius acadicus*	Strigiformes	Strigidae	8	
76	European starling	*Sturnus vulgaris*	Passeriformes	Sturnidae	1	
77	Gannet	*Sulidae sp*.	Suliformes	Sulidae	2	
78	Northern gannet	*Morus bassanus*	Suliformes	Sulidae	4	
79	American robin	*Turdus migratorius*	Passeriformes	Turdidae	4	
80	Robin	*Turdidae sp*.	Passeriformes	Turdidae	3	
	Unknown				89	
	Total bird samples				1798	6

**Fig 1:**
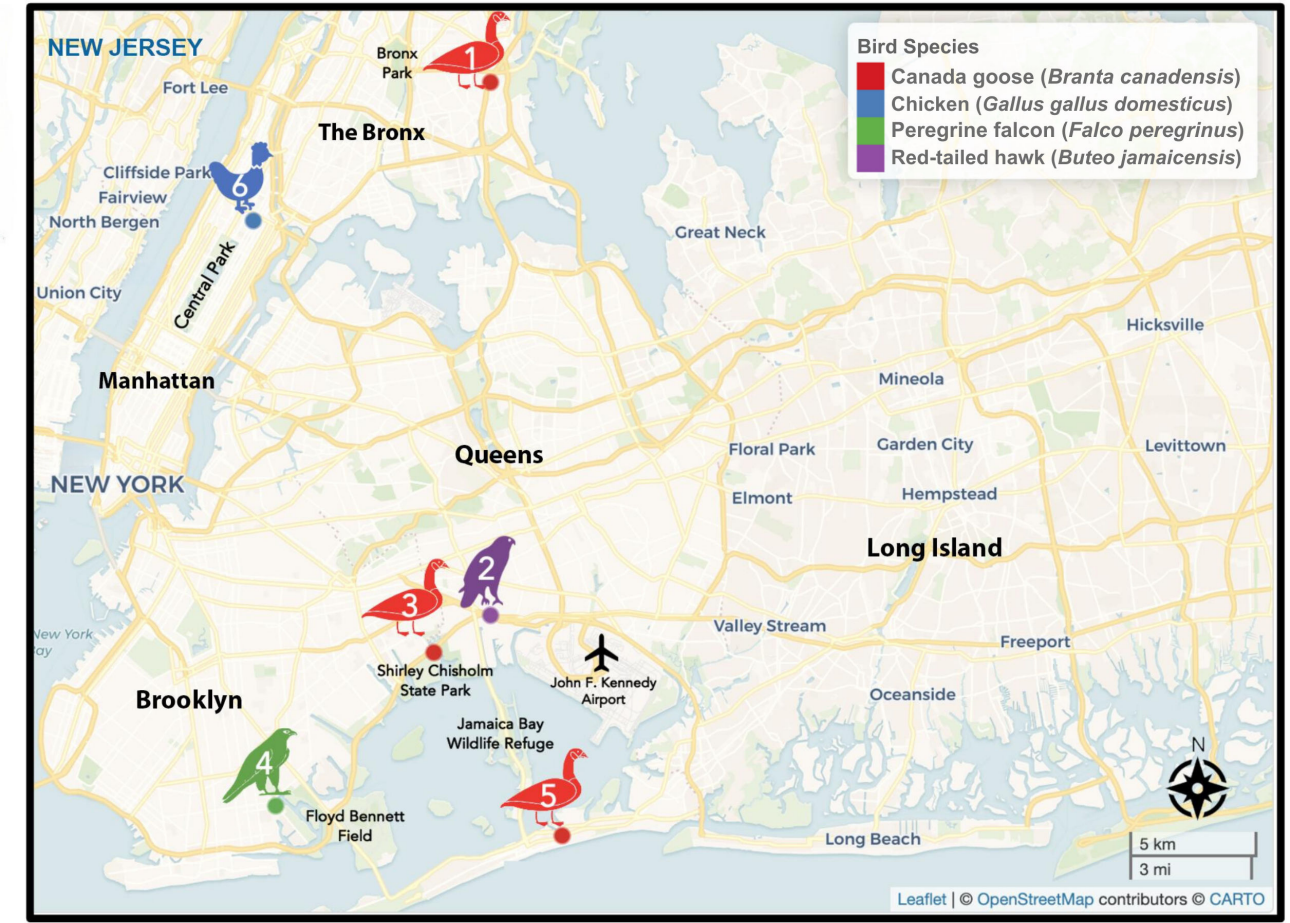
Map of location of birds that tested positive for highly pathogenic avian influenza H5N1 virus (HPAI H5N1) in New York City. The approximate locations are plotted based on geocoded addresses (latitude and longitude), providing a visual representation of affected areas. Major parks and natural areas are highlighted in green and labeled for context. The map was created using the leaflet package for mapping visualizations, with additional spatial data handling and esthetic enhancements performed using the sf, ggplot2, and dplyr packages in RStudio/Posit (Version 2023.09.1+494). The basemap was provided by CARTO, with data sourced from OpenStreetMap under the Open Data Commons Open Database License (ODbL) by the OpenStreetMap Foundation (OSMF).

Of these positive samples, the first [A/Canada goose/New York/NYCVH 22–6038/2022 (H5N1)] was collected from a Canada goose (*Branta canadensis*). This animal was initially found in Hutchinson River Parkway, in the Bronx, and died before the intake exam in August 2022. The next positive sample [A/red-tailed hawk/New York/NYCVH 22–8477/2022 (H5N1)] was derived in October 2022 from a red-tailed hawk (*Buteo jamaicensis*) that was found in close proximity to a major highway in Queens. The bird displayed neurological symptoms in the clinic. In December 2022, two birds found in Brooklyn tested positive for HPAI H5N1. One Canada goose [A/Canada goose/New York/NYCVH 22–9190/2022 (H5N1)] displayed neurological symptoms and cloudy eyes and one peregrine falcon (*Falco peregrinus*, strain name A/peregrine falcon/New York/NYCVH 160820/2022). The fifth sample [A/Canada goose/New York/NYCVH 23–453/2023 (H5N1)] came from a Canada goose found in February of 2023 in Queens. The sixth positive sample [A/chicken/New York/NYCVH 168127/2023 (H5N1)] was collected in April 2023 from a chicken (*Gallus gallus domesticus*) found in upper Manhattan (Fig. 1; Table 2). No additional positive samples/birds were detected from April 2023 to November 2023.

**TABLE 2:** Clinical and sampling information for wild birds positive on RT-PCR for highly pathogenic avian influenza virus (H5N1) in New York City from January 2022 to November 2023

Sample ID	Sample type	Species (common name)	Species (scientific name)	Location	Sampling date	Clinical signs
22–6038	Cloacal swab	Canada goose	*Branta canadensis*	Corner of Hutchinson River Pkwy. East and Wilkinson Avenue, Bronx, NY	August 24, 2022	Died before intake exam.
22–8477	Fecal	Red tailed hawk	*Buteo jamaicensis*	Belt Pkwy/Nassau Expressway, Queens, NY	October 22, 2022	Neurologic symptoms, loss of leg function, torticollis, and glottis wide open.
22–9190	Oropharyngeal swab	Canada goose	*Branta canadensis*	Fountain Avenue, Brooklyn, NY	December 3, 2022	Oculi uterque, eyes cloudy and occluded, head tremors, ataxia, and unable to stand.
160820	Oropharyngeal swab	Peregrine falcon	*Falco peregrinus*	Corner of Gerritsen Avenue and Avenue V, Brooklyn. NY	December 18, 2022	Unavailable
23–0453	Oropharyngeal swab	Canada goose	*Branta canadensis*	Beach 73^rd^ Street Averne, Queens, NY	February 1, 2023	Neurologic symptoms, ataxia, severe head tremors, partial torticollis, and labored breathing, oculi uterque, eyes cloudy and occluded.
168127	Fecal	Chicken	*Gallus gallus domesticus*	Corner of 120^th^ Street and 5th Avenue, Manhattan, NY	April 2, 2023	Unavailable

**Fig 2:**
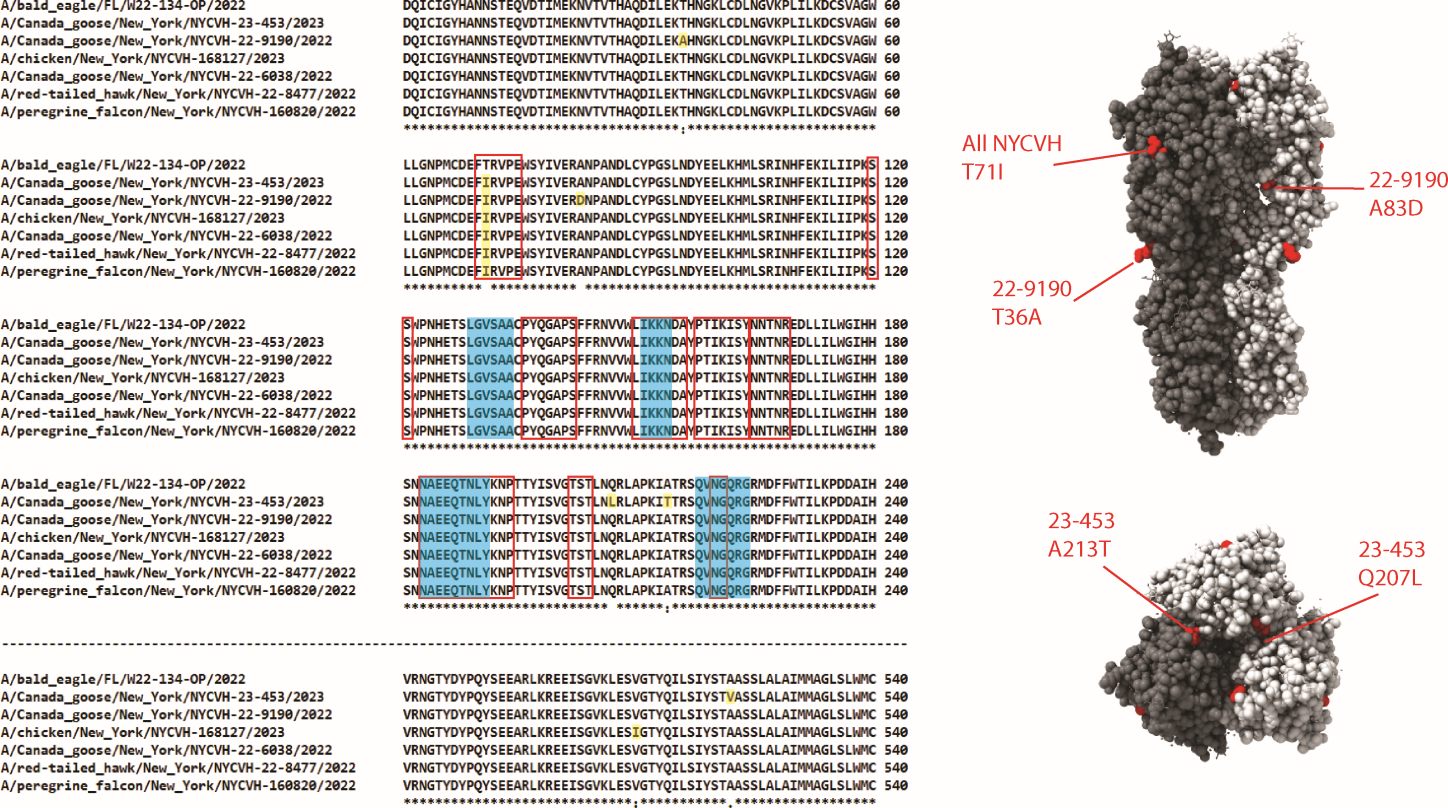
Amino acid sequence analysis of detected HPAI H5N1 strains. A multiple sequence alignment was performed using the HA sequences of NYCVH-detected strains and A/bald eagle/FL/W22-134-OP/2022 in Clustal Omega. Only areas of the alignment with amino acid differences are shown. Residues that were different in NYCVH strains compared to A/bald eagle/FL/W22-134-OP/2022 were highlighted in red on an H5N1 structure based on A/chicken/Vietnam/4/2003 (PDB #6VMZ) (42), visualized with UCSF ChimeraX. V510I and A522V are changes to positions not present in the PDB #6VMZ ectodomain structure. The receptor-binding site and antigenic sites are indicated by blue highlighting and red outline, respectively.

To further analyze our detected viruses, we performed a multiple sequence alignment of their amino acid sequences and mapped amino acid (AA) changes from the HPAI H5N1 strain A/bald eagle/FL/W22-134-OP/2022 (accession number UWI70064) (41) onto an HA structure from A/chicken/Vietnam/4/2003 (42) (Fig. 2). The detected AA differences mainly fell outside the receptor-binding site and antigenic sites of H5N1 (43, 44), except for T71I. Most differences were only found in one of our NYCVH strains, except for T71I, which was present in all NYCVH strains. It should be noted that isoleucine (I) was present at this position in all 50 strains used to construct our phylogenetic tree, and it is atypical for A/bald eagle/FL/W22-134-OP/2022 to have a threonine (T) at this position. To our knowledge, none of the amino acid changes relative to A/bald eagle/FL/W22-134-OP/2022 have specifically been implicated with increases in pathogenicity or mammalian adaptation.

Upon confirmation, detections were reported to the United States Department of Agriculture (USDA), and the associated original samples were transferred to Mount Sinai’s BSL3 +select agent facility (Emerging Pathogens Facility (EPF)/BSL-3 Biocontainment CoRE) for storage. Results were also discussed with the New York City Department of Health and Mental Hygiene as well as the Wild Bird Fund and the Animal Care Centers of New York City, following a previously developed internal and external communication strategy (45, 46). In brief, the strategy aimed to ensure prompt and informed decisions and that all participants, collaborators, and stakeholders are kept fully informed. Successful communication of science and public health messages is complex, and reaching potentially vulnerable audiences remains an important challenge. Our communication aimed to calm potential anxiety by providing information and instilling confidence and trust by addressing all questions to the best of our abilities. It has been noted that communications around emerging infectious disease can be improved when it comes from individuals inside the same community as those receiving the information, simply for the fact that they often share the same language, values, and beliefs (47). Therefore, it is incredibly important to ensure researchers involved in pandemic preparedness are committed to bidirectional communication, listening and serving the needs of the community. To reach the scientific community and general public alike, involved students shared their results in multiple languages and through multiple channels. These range from live virtual events and talks at community boards to in-person symposia and presentations at scientific conferences. Results were also presented to the public at three student research symposia, including the New York City Virus Hunters Symposium on 31 May 2023.

### Phylogenetic analysis of detected HPAI H5N1 viral genomes

**Fig 3:**
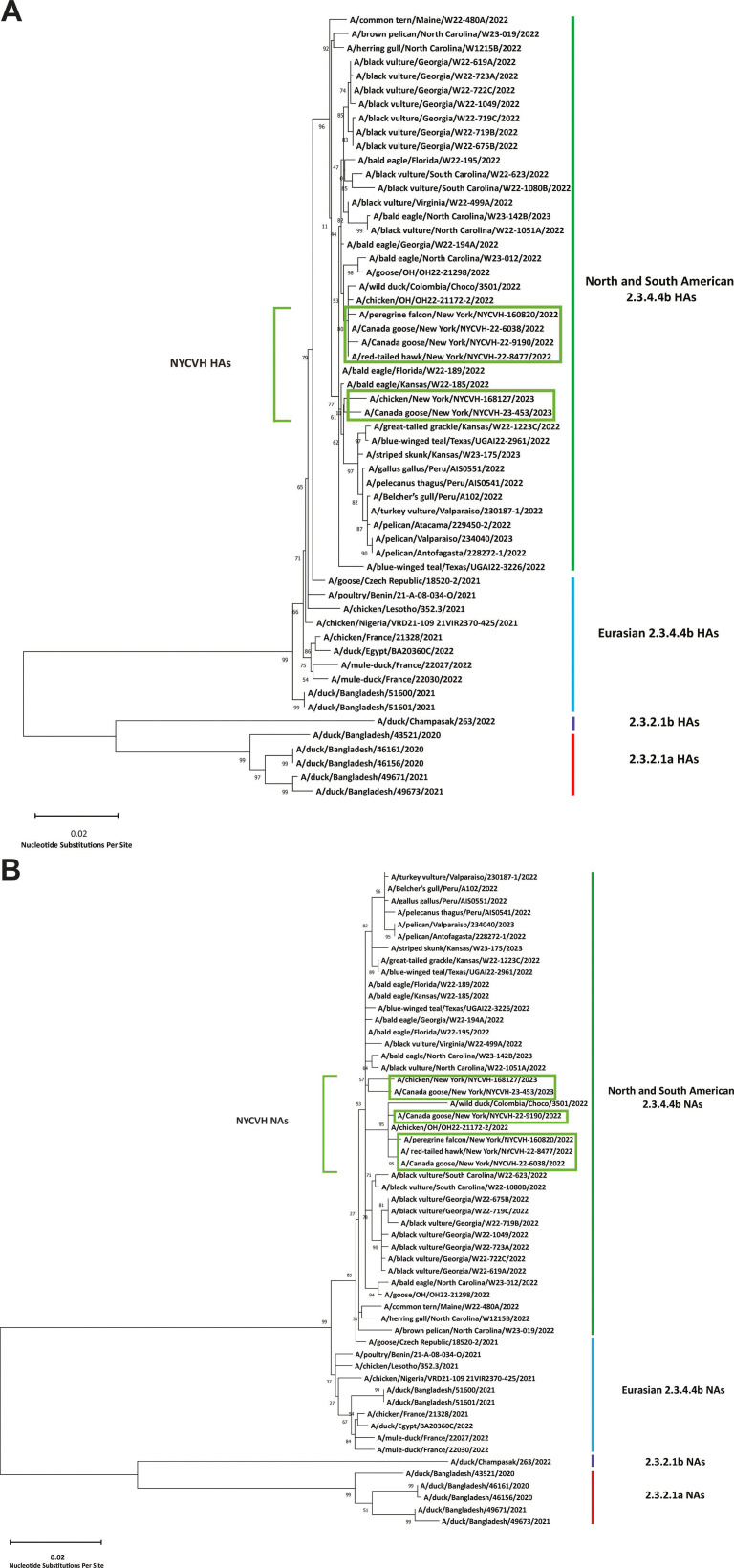
Phylogenetic tree of HA and NA genes of the detected viruses in comparison to sequences from GenBank. HA (**A**) and NA (**B**) gene sequences from 50 strains of H5N1 influenza virus were randomly selected from all available strains collected between 1 January 2020 and 26 October 2023 (NCBI influenza virus database, https://www.ncbi.nlm.nih.gov/genomes/FLU/Database/nph-select.cgi). The 50 randomly selected H5N1 sequences and the six NYCVH H5N1 sequences were used to create a phylogenetic tree in MEGA 11 (https://www.megasoftware.net) using the maximum likelihood method and Tamura–Nei model (48) with a bootstrap test (*n* = 100). Bootstrap support values are shown by branch nodes. The scale bar indicates nucleotide substitutions per site.

Phylogenetic analysis of the six viral genomes and genotype assignment was performed. The H5 and N1 genes of all six viruses were all typical of the currently circulating 2.3.4.4b clade in the Americas. HA sequences of A/Canada goose/New York/NYCVH 22–6038/2022, A/red-tailed hawk/New York/NYCVH 22–8477/2022, A/Canada goose/New York/NYCVH 22–9190/2022, and A/peregrine falcon/New York/NYCVH 160820/2022 were clustered closely together in a tree constructed of 50 HA sequences randomly selected from a list of all available H5N1 strains collected since 1 January 2020 on NCBI’s influenza virus database, downloaded on 26 October 2023 (Fig. 3). They also cluster with contemporary H5 sequences from 2022 from Ohio, North Carolina, and also Colombia. Similarly, their NA sequences cluster together next to the NA sequences of the Ohio and Colombia isolates for which the HAs cluster as well. The two 2023 sequences A/chicken/New York/NYCVH 168127/2023 and A/Canada goose/New York/NYCVH 23–453/2023 are clustering together as well and form their own branch close to a cluster of sequences from 2022 and 2023 North and South American isolates. The NA sequences of these two viruses cluster together but are also located closely to many different isolates from both North and South America.

To identify the genotypes of internal genes, we used a script provided by Youk *et al*. (16) that allows for classification of segments into lineages and determines a genotype based on the genomic segment composition of a virus. We compared our virus sequences with available full-length genome sequences from the New York State, New Jersey, and Connecticut areas surrounding New York City where many infections were detected (Fig. S1). All detected viruses were re-assortant viruses between the Eurasian (EA) and American (AM) lineages. All HA and NA segments were of course from the EA lineage, but segments encoding for internal proteins differed. A/Canada goose/New York/NYCVH 22–6038/2022, A/red-tailed hawk/New York/NYCVH 22–8477/2022, A/Canada goose/New York/NYCVH 22–9190/2022, and A/peregrine falcon/New York/NYCVH 160820/2022 were all determined to be genotype B1.3 with AM lineage polymerase and NP segments, and all other segments were from the EA lineage (Table 3). B1.3 lineage viruses were also found in New York State and neighboring states (New Jersey and Connecticut) during our observation period (Table 4). The more recent A/chicken/New York/NYCVH 168127/2023 and A/Canada goose/New York/NYCVH 23–453/2023 viruses belonged to lineage B3.3, a lineage also detected in New York State in a turkey vulture in April 2023. This lineage features PB2, PB1, NP, and NS segments from the AM lineage, while PA, HA, NA, and M segments are derived from the EA lineage.

**TABLE 3:** Genotypes of the detected H5N1 strains^*a*^

Strain	Genotype	PB2	PB1	PA	HA	NP	NA	M	NS
A/red-tailed hawk/New York/NYCVH 22–8477/2022	B1.3	am1.3	am1.3	am1.2	ea1	am1.2	ea1	ea1	ea1
A/Canada goose/New York/NYCVH 22–6038/2022	B1.3	am1.3	am1.3	am1.2	ea1	am1.2	ea1	ea1	ea1
A/Canada goose/New York/NYCVH 22–9190/2022	B1.3	am1.3	am1.3	am1.2	ea1	am1.2	ea1	ea1	ea1
A/peregrine falcon/New York/NYCVH 160820/2022	B1.3	am1.3	am1.3	am1.2	ea1	am1.2	ea1	ea1	ea1
A/Canada goose/New York/NYCVH 23–453/2023	B3.2	am2.1	am1.2	ea1	ea1	am1.4.1	ea1	ea1	am1.1
A/chicken/New York/NYCVH 168127/2023	B3.3	am2.2	am1.4	ea1	ea1	am1.4.1	ea1	ea1	am1.1

^*a*^ American lineages are abbreviated as “am,” and Eurasian lineages are abbreviated as “ea.” Influenza virus gene segments are abbreviated as follows: polymerase basic 2 (PB2), polymerase basic 1 (PB1), polymerase acidic (PA), hemagglutinin (HA), nucleoprotein (NP), neuraminidase (NA), matrix (M), and nonstructural protein (NS).

**TABLE 4:** Genotypes of strains detected in New York, New Jersey, and Connecticut from August 2022–April 2023^*a*^

Collection date	Strain	Genotype	PB2	PB1	PA	HA	NP	NA	M	NS
10/11/2022	A/domestic duck/New Jersey/22–032412-001-original/2022	B1.1	am1.1	am1.1	ea1	ea1	am1.2	ea1	ea1	ea1
10/21/2022	A/African goose/New Jersey/22–033679-001-original/2022	B1.3	am1.3	am1.3	am1.2	ea1	am1.2	ea1	ea1	ea1
10/28/2022	A/wild turkey/New York/22–034864-002-original/2022	B1.3	am1.3	am1.3	am1.2	ea1	am1.2	ea1	ea1	ea1
11/1/2022	A/chicken/New Jersey/22–035003-009-original/2022	B1.3	am1.3	am1.3	am1.2	ea1	am1.2	ea1	ea1	ea1
11/3/2022	A/chicken/New York/22–035475-001-original/2022	B2.2	am1.2	ea1	ea1	ea1	am1.1	ea1	ea1	am1.2
11/3/2022	A/chicken/New York/22–035476-001-original/2022	B2.2	am1.2	ea1	ea1	ea1	am1.1	ea1	ea1	am1.2
11/7/2022	A/Muscovy duck/New York/22–036304-003-original/2022	B1.3	am1.3	am1.3	am1.2	ea1	am1.2	ea1	ea1	ea1
11/7/2022	A/chicken/New York/22–036304-007-original/2022	B1.3	am1.3	am1.3	am1.2	ea1	am1.2	ea1	ea1	ea1
2/8/2023	A/American crow/New York/23–004806-001-original/2023	B3.5	am3.2	am1.2	ea1	ea1	am1.4.1	ea1	ea1	am1.1
2/10/2023	A/great horned owl/New York/23–005127-001-original/2023	B1.2	am1.2	ea1	ea1	ea1	am1.1	ea1	am1	am1.1
2/11/2023	A/red-tailed hawk/New York/23–005536-001-original/2023	B2.2	am1.2	ea1	ea1	ea1	am1.1	ea1	ea1	am1.2
2/13/2023	A/American crow/New York/23–005128-001-original/2023	B3.5	am3.2	am1.2	ea1	ea1	am1.4.1	ea1	ea1	am1.1
2/13/2023	A/peregrine falcon/New York/23–005700-001-original/2023	B1.3	am1.3	am1.3	am1.2	ea1	am1.2	ea1	ea1	ea1
2/15/2023	A/Canada goose/New York/23–005698-001-original/2023	B1.2	am1.1	am1.1	am1	ea1	am1.2	ea1	ea1	ea1
2/15/2023	A/Canada goose/New York/23–005699-001-original/2023	B1.2	am1.1	am1.1	am1	ea1	am1.2	ea1	ea1	ea1
2/15/2023	A/Canada goose/New York/23–005699-001-original/2023	B1.2	am1.1	am1.1	am1	ea1	am1.2	ea1	ea1	ea1
2/20/2023	A/American crow/New York/23–005695-001-original/2023	B3.5	am3.2	am1.2	ea1	ea1	am1.4.1	ea1	ea1	am1.1
2/22/2023	A/Canada goose/New York/23–006363-001-original/2023	B3.5	am3.2	am1.2	ea1	ea1	am1.4.1	ea1	ea1	am1.1
2/27/2023	A/American crow/New York/23–006663-001-original/2023	B3.5	am3.2	am1.2	ea1	ea1	am1.4.1	ea1	ea1	am1.1
2/27/2023	A/Canada goose/New York/23–006664-001-original/2023	B3.5	am3.2	am1.2	ea1	ea1	am1.4.1	ea1	ea1	am1.1
3/6/2023	A/Canada goose/New York/23–008112-001-original/2023	B1.2	am1.1	am1.1	am1	ea1	am1.2	ea1	ea1	ea1
3/15/2023	A/American crow/New York/23–009037-001-original/2023	B3.5	am3.2	am1.2	ea1	ea1	am1.4.1	ea1	ea1	am1.1
3/16/2023	A/peregrine falcon/New York/23–009036-001-original/2023	B3.5	am3.2	am1.2	ea1	ea1	am1.4.1	ea1	ea1	am1.1
3/17/2023	A/American crow/New York/23–009035-001-original/2023	B3.5	am3.2	am1.2	ea1	ea1	am1.4.1	ea1	ea1	am1.1
3/23/2023	A/red-tailed hawk/Connecticut/23–009886-001-original/2023	B1.3	am1.3	am1.3	am1.2	ea1	am1.2	ea1	ea1	ea1
3/23/2023	A/red-tailed hawk/Connecticut/23–009880-001-original/2023	B1.3	am1.3	am1.3	am1.2	ea1	am1.2	ea1	ea1	ea1
4/6/2023	A/turkey vulture/New York/23–011005-001-original/2023	B3.3	am2.2	am1.4	ea1	ea1	am1.4.1	ea1	ea1	am1.1
4/13/2023	A/red-shouldered hawk/New York/23–012563-001-original/2023	B3.2	am2.1	am1.2	ea1	ea1	am1.4.1	ea1	ea1	am1.1
4/21/2023	A/Canada goose/New York/23–013244-001-original/2023	B1.3	am1.3	am1.3	am1.2	ea1	am1.2	ea1	ea1	ea1
4/27/2023	A/American crow/New York/23–014010-001-original/2023	B3.5	am3.2	am1.2	ea1	ea1	am1.4.1	ea1	ea1	am1.1
4/27/2023	A/Canada goose/New York/23–014011-001-original/2023	B1.3	am1.3	am1.3	am1.2	ea1	am1.2	ea1	ea1	ea1

^*a*^ American lineages are abbreviated as “am,” and Eurasian lineages are abbreviated as “ea.” Influenza virus gene segments are abbreviated as follows: polymerase basic 2 (PB2), polymerase basic 1 (PB1), polymerase acidic (PA), hemagglutinin (HA), nucleoprotein (NP), neuraminidase (NA), matrix (M), and nonstructural protein (NS).

## DISCUSSION

The recent spread of the panzootic clade 2.3.4.4b H5N1 across the globe has caused significant damage to wild bird populations and to the poultry industry (16, 41, 49, 50). Spillovers into mammals (including cattle) have caused concerns about mammalian adaptation of this clade. However, despite the extensive spread of the clade 2.3.4.4b H5N1 virus and likely significant exposure of humans to it (hunters, poultry farmers, etc.), human infections have so far been rare, with only two known severe cases in the Americas (29, 30) and a small number of cases in Asia (31). Avian influenza virus surveillance is often carried out in wild birds in rural areas, through hunter programs as well as domestic poultry operations. However, surveillance systems to detect the virus in urban wild birds are often absent. Despite that, many bird species inhabit or temporarily visit urban areas, which in many cases have ample green space as well as aquatic habitats for waterfowl. This is exemplified by the long list of species sampled in this study. Our study focused on this urban space using two sample streams, namely, samples from animal rehabilitation centers (Wild Bird Fund and Animal Care Centers of New York City) and environmental fecal samples sourced via a citizen/community science project (New York City Virus Hunters). Including the community in viral surveillance in a safe way generates interest and an understanding of the topic in the population, which is important given the science skepticism that has come to light through the coronavirus disease 2019 (COVID-19) pandemic (51, 52).

Our work identified six HPAI H5N1 viruses in 1,927 samples (corresponding to at least 895 birds). These viruses were found in species known to be susceptible for H5N1 infection. Based on infection patterns in our area, we did expect to find HPAI H5N1 virus in Canada geese (which are highly susceptible to H5N1 infections (53, 54)) as well as in raptors (peregrine falcon and red-tailed hawk), which often get infected when feeding on infected prey or carcasses. While H5N1 is known to infect chickens, it was somewhat unexpected to receive samples from a chicken found in Marcus Garvey Park in Manhattan. Almost all our other samples from chickens were from birds in captivity. It remains unclear if the chicken in Marcus Garvey Park was intentionally released or escaped from captivity elsewhere, as does the context in which they became infected (in captivity or after release). It is important to state that all six positive samples came from either the Wild Bird Fund or the Animal Care Centers of New York City, stressing the important role that urban wildlife rehabilitation centers can play in urban viral surveillance efforts. The detected HA and NA sequences clustered with other H5 and N1 sequences from North and South American clade 2.3.4.4b H5N1 viruses circulating at approximately the same time, and they belonged to two different genotypes, which are both reassortants between the Eurasian 2.3.4.4b H5N1 and American avian influenza viruses. It has recently been shown that these reassortants can have increased pathogenicity in mammals as compared to the full Eurasian genotype of 2.3.4.4b H5N1 (41). The genotypes of our NYCVH-detected HPAI H5N1 viruses have also been detected in the region (defined as the states of New York, New Jersey, and Connecticut) during the same time period. Of note, while many infections in mammals have been reported in the Americas with severe (and often neurological) symptoms and outcomes, most have been “dead end” infections in scavengers or predatory animals that presumably fed on infected birds or bird carcasses (20–22). However, mammal-to-mammal transmission is suspected in several outbreaks in fur farms in Europe and in marine mammals in South America (26–28), and recent cases in cows in several US states and in a goat have raised concerns.

Our study shows that clade 2.3.4.4b H5N1 highly pathogenic avian influenza virus can be present in birds that migrate through or live in urban centers. This highlights the importance of viral surveillance at the urban animal–human interface in which wild animals may potentially interact with a high-density population of humans and their pets. Humans may interact with infected birds directly (handling an injured bird) or indirectly (e.g., by coming in contact with feces or contaminated water in parks). Pets including cats and dogs are susceptible to HPAI H5N1, and transmissions from birds to both pet species have happened via contact with infected birds or bird carcasses—scenarios which could occur in urban green spaces where pets are frequently taken (32–35, 55). Our study highlights these risks. However, it needs to be emphasized that a very small number of birds were found positive. Of note, the low percentage of positive animals could also be due to the sensitivity of the screening pipeline used, and other assays or tests may produce a higher number of positives.

An important aspect of our work is to involve the population and all stakeholders in surveillance efforts and communicate findings and risks efficiently. For this, we have shared and discussed our results with the New York City Department of Health and Mental Hygiene, and we have worked out a communication strategy. Junior scientists from the New York City Virus Hunters Program have also shared the results of our study with the public during our annual symposium in May 2023. We believe it is important for the public to understand that HPAI H5N1 may be present in birds, as well as their feces and other secretions in urban spaces, that sick or weirdly behaving birds (or other wildlife) should be reported to the authorities and only be handled by professionals in proper personal protective equipment, and that pets should be kept away from urban wildlife. Furthermore, it is important for physicians in urban centers to know about the potential presence of HPAI H5N1 and be aware that atypical influenza cases in humans may be caused by avian influenza viruses. So far, studies suggest that North American clade 2.3.4.4b viruses are susceptible to all classes of influenza drugs that are available as treatment options (41).

In summary, through a science outreach and community science project, we found clade 2.3.4.4b H5N1 highly pathogenic avian influenza viruses in New York City birds. The presence of the virus poses a low but non-zero risk for humans and pets, and more awareness about the presence of this virus at the urban animal–human interface is needed.

## MATERIALS AND METHODS

### Sample collection

No birds were killed for the purposes of this study. Work at Mount Sinai was approved by the Icahn School of Medicine at Mount Sinai Institutional Animal Care and Use Committee (IPROTO202300000038). Sampling in New York City parks was permitted by the New York City Parks and Recreation. The WBF operates under a Department of Environmental Conservation license, and both WBF and ACC operate under a U.S. Fish & Wildlife Service Migratory Bird permit. Fecal and swab samples from live, sick, and recently deceased or euthanized birds were provided by the WBF and ACC and were collected by veterinarians or licensed veterinary technicians as part of standard veterinary care of the birds. Water samples (3 mL each) were collected at the WBF’s indoor waterfowl rehabilitation pool, using sterile pipettes and stored individually in cryotubes. Sampling focused on aquatic birds, particularly Anseriformes (including *Anas* ducks, geese, and swans), Ciconiiformes (including gulls, cormorants, and shorebirds), and raptors (including hawks, eagles, and falcons). All live bird sampling procedures were performed by New York State licensed wildlife rehabilitators employed by the WBF or ACC. Fecal, oropharyngeal, and cloacal swabs were collected from each bird using sterile flocked nylon-tipped swabs and stored individually in cryovials containing either MicroTest viral transport medium (Thermo Scientific, USA) or a medium containing 50% phosphate-buffered saline and 50% glycerol, supplemented with 1% antibiotic–antimycotic 100X (Gibco, Thermo Scientific, USA). Samples were kept at 4°C for up to 4 hours, stored at −20°C for up to 7 days, and then stored at −80°C . The cold chain was maintained throughout delivery of samples to the laboratory. Environmental fecal samples that appeared fresh (still moist) were collected opportunistically in urban parks and greenspaces where birds were observed congregating, sacrificing the specific identity of the birds being sampled. Environmental fecal samples included in this study were collected on the following locations and dates (12 sampling field trips total): Manhattan, New York: Central Park (May 2022 and January and November 2023), Riverside Park (April and June 2023), Tompkins Square Park ( April 22 and May 2023), and South Bronx, New York: Saint Mary’s Park (October 2023). For each location, samples were obtained over a wide area of interest and not a single point. To avoid sampling of the same bird more than once, samples were collected with a minimum distance of 20 cm between each other. A transect sampling strategy was employed when sampling around bodies of water, like city ponds. All samples were collected and preserved in the same manner as those collected from live birds. When possible, samples were collected avoiding visible uric acid and soil to prevent potential contamination with PCR inhibitors.

### RNA extraction and RT-PCR

Fecal samples were diluted in phosphate-buffered saline, pH 7.4 (1X, Thermo Scientific, USA) for processing. Suspended fecal samples, oropharyngeal swabs and cloacal swabs, were centrifuged at 4,000  ×  g for 15  min, and viral RNA was extracted from each supernatant using the QIAamp Viral RNA minikit (Qiagen, USA) according to the manufacturer’s instructions. The Stool Total RNA purification kit (Norgen Biotek Corporation, Canada) was also used to extract RNA from fecal samples. Samples collected from the same bird were not pooled. The conventional two-step reverse transcriptase polymerase chain reaction (RT-PCR) was employed using the Invitrogen SuperScript IV first-strand synthesis system (Thermo Scientific, USA) for cDNA synthesis and DreamTaq Green PCR Master Mix (2X) (Thermo Scientific, USA) for RT-PCR. First, cDNA was synthesized using a minimum of 250  ng of RNA at 55°C for 10  min using a previously described universal primer [Uni12, *AGCAAAAGCAGG* (56)]. Then, cDNA was amplified using previously described primers for HPAI H5N1 surveillance that target the nucleoprotein [NP, NP1200 forward, *CAGRTACTGGGCHATAAGRAC* and NP1529 reverse, *GCATTGTCTCCGAAGAAATAAG* (57)], matrix [M, M52C forward, *CTTCTAACCGAGGTCGAAACG* and M253R reverse, *AGGGCATTTTGGACAAAKCGTCTA (57*)], and hemagglutinin [H5, H5.2344–1673 forward, *TACCAAATAYTGTCAATTTATTCAAC* and H5.2344–1749 reverse, *GTAAYGACCCRTTRGARCACATCC (58*)] genes. Primers for HA (H5) were included for prompt identification of HPAI H5N1 viruses, facilitating quick notification of partner organizations as necessary to handle infected birds. Cycling conditions for the multiplex PCR consisted of a pre-denaturation step at 95°C for 1  min, followed by 30 cycles of denaturation at 95°C for 1  min, annealing at 45°C for 30  s, and extension at 72°C for 30  s, with a final extension step at 72°C for 5  min. PCR amplicons were visualized with SYBR Safe DNA Gel Stain in 2% Ultra-Pure Agarose (Thermo Scientific, USA). DNA bands were excised and purified using the QIAquick Gel Extraction Kit (Qiagen, USA) and sent for commercial Sanger sequencing (through Genewiz, New Jersey facility) to confirm the identity of samples that screened positive. Samples that screened positive for H5 HA and also were identified as H5 by Sanger sequencing (to exclude false positives) were reported to the USDA. The remaining sample material was moved to Mount Sinai’s select agent facility for storage.

### Next-generation sequencing

Samples which tested positive for H5, NP, and M by PCR were then used for next-generation sequencing. RT-PCR products were quantified on a Qubit 4 Fluorometer using HS DNA reagents (Invitrogen). A volume of 3.5 µL of the cDNA product was used in a 50 µL PCR reaction with Phusion™ High-Fidelity DNA Polymerase (2 U/µL) (ThermoFisher). Three universal influenza A primers at 0.20 µM concentration were used in the PCR. Commonuni13 (GCCGGAGCTCTGCAGATATCAGTAGAAACAAGG), Commonuni12G (GCCGGAGCTCTGCAGATATCAGCGAAAGCAGG), and Commonuni12A (GCCGGAGCTCTGCAGATATCAGCA AAAGCAGG)*,* 0.2 µM dNTPs, and 1X HF buffer were also components of the reaction. Single-reaction multiplex PCR was performed for sample amplification. Amplification occurred under the following cycling parameters: samples were initially denatured at 94°C for 2 minutes and then underwent five cycles of 94°C for 30 seconds, 45°C for 30 seconds, and 60°C for 3 minutes, followed by cycling parameters 31 cycles of 94°C for 30 seconds, 57°C for 30 seconds, and 68°C for 3 minutes and then a 4°C hold. PCR products underwent DNA purification and size selection using AMpure XP beads. Library preparation was performed using the Nextera XT DNA Library Preparation Kit (Illumnia, CA, USA) following the manufacturer’s protocol to generate multiplex paired-end sequencing libraries. Post-fragmentation automated electrophoresis was performed using the Tape Station 1450 (Agilent Technologies). Sample libraries were quantified using a Qubit 4 Fluorometer (Invitrogen). Sample molarity was determined according to the following formula:


$$\frac{\left(Sample\;concentration\;in\;ng/\mu l\right)}{\left(Average\;number\;of\;bp\;x\;660\;g/mols\right)}\;x\;10^{6}$$

Samples were pooled using Illumina’s pooling calculator in equimolar amounts, creating a paired-end fragmented library pool in which each sample is represented with unique indices. Upon pooling and diluting samples to 4 nM using 8.5 pH 10 mM tris(hydroxymethyl)aminomethane (Tris)-HCl with 0.1% Tween 20 following “Protocol A: Standard Normalization method” from the “illlumina MiSeq System: Denature and Dilute Libraries Guide”. Samples were sequenced using a MiSeq device (Illumnia, CA, USA) at a final concentration of 12 pM with a 15% PhiX spike in. After the 2 × 300 bp MiSeq paired-end sequencing run, the instrument performed base-calling on the data and collected reads with matching indices to generate paired-end forward and reverse read Illumina FASTQ files for each sample.

Illumina reads in the form of FASTQ files are produced by the MiSeq with adapter sequences on the 5’ and 3’ ends for sample identification; therefore, the first step in analysis was to remove the non-viral DNA. FASTQ files were uploaded to the Galaxy web platform, and several tools available through the *usegalaxy.org* public server (59) were used for genome assembly. Cutadapt (Martin) was used to remove adapter sequences and to generate FASTA files for analysis. Metagenome *de novo* assembly was performed using the metaSPAdes approach. Nucleotide to protein BLAST (NCBI) was performed on generated sequences to confirm the identity and relevance (60). Influenza A virus segments were identified by NCBI Flu annotator (61). Nucleotide to nucleotide BLAST was run to determine reference sequences. FASTA files were then indexed and mapped to the reference genome using BWA-MEM2 (62) and *Simple Illumina* parameters. BWA-MEM2 produces sorted and indexed BAM files. To account for potential laboratory-derived influenza virus contaminants that could alter the final consensus sequences, we performed the same analysis on a filtered read set, generated by taking the unaligned reads left over after aligning the data to the A/Puerto Rico/8/1934 reference genome (NCBI Taxonomy ID id183764) using Bowtie2 (63). To ensure only reads definitively derived from known contaminants were excluded and no reads derived from influenza virus from the sample were discarded, we adjusted the parameters of the alignment algorithm. To increase strictness, the alignment was run in the end-to-end mode; the mismatch penalties, gap opening, and extension penalties were increased; and the maximum seed substring length allowing for zero sequence mismatches in the seed alignment during multiseed alignment was used. Final analysis and consensus sequences were produced using the indexed BAM file and iVar Consensus (64) and Geneious. The antigenic genotypes of the consensuses sequences were confirmed using the NCBI Flu annotator. Nucleotide to protein BLAST (NCBI) was performed on generated sequences to confirm identity and relevance. Sequence relevance is determined by the location of isolation and date of isolation in terms of the closest reference sequence, as well as its similarity to existing reference sequences for HPAI H5N1 viruses from 2022 onward.

### Phylogenetic analysis

A phylogenetic analysis comparing the sequences obtained from next-generation sequencing with other recent H5N1 sequences was performed. All available HA and NA sequences from H5N1 strains collected since 1 January 2020 were downloaded as FASTA files from the NCBI influenza virus database (https://www.ncbi.nlm.nih.gov/genomes/FLU/Database/nph-select.cgi) on 26 October 2023. Fifty pairs of HA and NA sequences (from the same influenza virus strain) were randomly selected for inclusion in the phylogenetic tree. In addition to our sequences, the following sequences were used: A/black vulture/Georgia/W22-1049/2022: WEV84579, WEV84580; A/bald eagle/Florida/W22-189/2022: OP221398, OP221399; A/mule-duck/France/22030/2022: OQ632831, OQ632862; A/duck/Bangladesh/49673/2021: OP023902, OP023904; A/duck/Egypt/BA20360C/2022: OP590397, OP590399; A/duck/Bangladesh/49671/2021: OP023710, OP023712; A/black vulture/North Carolina/W22-1051A/2022: OQ694936, OQ694937; A/goose/Czech Republic/18520-2/2021: OL638145, OL638147; A/chicken/Lesotho/352.3/2021: OL477524, OL477526; A/turkey vulture/Valparaiso/230187-1/2022: OR125345, OR125347; A/black vulture/Georgia/W22-719C/2022: OQ584791, OQ584792; A/striped skunk/Kansas/W23-175/2023: OQ954544, OQ954545; A/duck/Bangladesh/51601/2021: OP030702, OP030704; A/blue-winged teal/Texas/UGAI22-3226/2022: OQ733076, OQ733077; A/blue-winged teal/Texas/UGAI22-2961/2022: OQ733108, OQ733109; A/pelican/Atacama/229450-2/2022: OR125340, OR125342; A/black vulture/Georgia/W22-723A/2022: OQ600260, OQ584498; A/chicken/Nigeria/VRD21-109_21VIR2370-425/2021: MW961460, MW961462; A/black vulture/South Carolina/W22-1080B/2022: OQ694870, OQ694871; A/pelecanus thagus/Peru/AIS0541/2022: OQ547335, OQ547337; A/black vulture/Georgia/W22-675B/2022: OQ584544, OQ584545; A/black vulture/Georgia/W22-619A/2022: OQ584552, OQ584553; A/black vulture/South Carolina/W22-623/2022: OQ584575, OQ584576; A/mule-duck/France/22027/2022: OQ632829, OQ632861; A/chicken/France/21328/2021: OQ632895, OQ632900; A/great-tailed grackle/Kansas/W22-1223C/2022: OQ734910, OQ734911; A/pelican/Antofagasta/228272-1/2022: OR125399, OR125401; A/black vulture/Virginia/W22-499A/2022: OP377388, OP377389; A/pelican/Valparaiso/234040/2023: OR125162, OR125164; A/duck/Bangladesh/43521/2020: MW466215, MW466211; A/black vulture/Georgia/W22-722C/2022: OQ584606, OQ584607; A/wild duck/Colombia/Choco/3501/2022: OQ683498, OQ683500; A/duck/Bangladesh/46156/2020: OM938314, OM938316; A/goose/OH/OH22-21298/2022: OR136609, OR136611; A/duck/Bangladesh/46161/2020: OM938292, OM938294; A/herring gull/North Carolina/W1215B/2022: OQ734937, OQ734938; A/bald eagle/North Carolina/W23-142B/2023: OQ732988, OQ732989; A/brown pelican/North Carolina/W23-019/2022: OQ734918, OQ734919; A/poultry/Benin/21-A-08–034-O/2021: ON870434, ON943071; A/bald eagle/Florida/W22-195/2022: OP221327, OP221328; A/bald eagle/Georgia/W22-194A/2022: OP221382, OP221383; A/duck/Bangladesh/51600/2021: OP030710, OP030712; A/gallus gallus/Peru/AIS0551/2022: OQ547415, OQ547417: A/bald eagle/Kansas/W22-185/2022: OP377646, OP377647; A/bald eagle/North Carolina/W23-012/2022: OQ982396, OQ982397; A/common tern/Maine/W22-480A/2022: OP377502, OP377503; A/black vulture/Georgia/W22-719B/2022: OQ737753, OQ737754; A/chicken/OH/OH22-21172-2/2022: OR136572, OR136574; A/duck/Champasak/263/2022: OR105066, OR105068; A/Belcher’s_gull/Peru/A102/2022: OQ747759, OQ747766. The sequences were aligned with MUSCLE (65) and were manually trimmed to remove noncoding regions before and after the protein sequence. The tree was created in MEGA 11 (https://www.megasoftware.net) using the maximum likelihood method (48) with a bootstrap test (*n* = 100). Trees were generated with the neighbor-joining and BioNJ algorithms applied to a matrix of pairwise distances created using the Tamura–Nei model, and the topology with the superior log-likelihood value was selected. Multiple sequence alignment of amino acid sequences was performed with Clustal Omega v1.2.4. The protein structure was visualized with UCSF ChimeraX, using the publicly available H5 structure #6VMZ (42).

### Genotyping and survey of HPAI H5N1 detected in New York, New Jersey, and Connecticut

Genotyping was carried out according to a method and data pipeline established by Youk *et al*. (16) (https://github.com/USDA-VS/GenoFLU). HPAI H5N1 sequences from samples collected in New York, Connecticut, and New Jersey between August 2022 and April 2023 were downloaded on 26 March 2024 from the Global Initiative for Sharing All Influenza Data (GISAID). HPAI H5N1 detection data for August 2022–April 2023 were downloaded on 24 March 2024 from the United States Department of Agriculture, Animal and Plant Health Inspection Service (USDA APHIS) database on wild bird HPAI H5N1 detections. The following sequences were used: A/domestic duck/New Jersey/22–032412-001-original/2022: EPI2264144, EPI2264145, EPI2264143, EPI2264147, EPI2264140, EPI2264146, EPI2264142, and EPI2264141; A/African goose/New Jersey/22–033679-001-original/2022: EPI2263775, EPI2263776, EPI2263774, EPI2263778, EPI2263771, EPI2263777, EPI2263773, and EPI2263772; A/wild turkey/New York/22–034864-002-original/2022: EPI2263599, EPI2263600, EPI2263598, EPI2263602, EPI2263595, EPI2263601, EPI2263597, and EPI2263596; A/chicken/New Jersey/22–035003-009-original/2022: EPI2263463, EPI2263464, EPI2263462, EPI2263466, EPI2263459, EPI2263465, EPI2263461, and EPI2263460; A/chicken/New York/22–035475-001-original/2022: EPI2260707, EPI2260708, EPI2260706, EPI2260710, EPI2260703, EPI2260709, EPI2260705, and EPI2260704; A/chicken/New York/22–035476-001-original/2022: EPI2260715, EPI2260716, EPI2260714, EPI2260718, EPI2260711, EPI2260717, EPI2260713, and EPI2260712; A/Muscovy duck/New_York/22–036304-003-original/2022: EPI2260795, EPI2260796, EPI2260794, EPI2260798, EPI2260791, EPI2260797, EPI2260793, and EPI2260792; A/chicken/New York/22–036304-007-original/2022: EPI2260803, EPI2260804, EPI2260802, EPI2260806, EPI2260799, EPI2260805, EPI2260801, and EPI2260800; A/American crow/New York/23–004806-001-original/2022: EPI2613579, EPI2613580, EPI2613578, EPI2613582, EPI2613575, EPI261358, EPI2613577, and EPI2613576; A/great horned owl/New York/23–005127-001-original/2023: EPI2613603, EPI2613604, EPI2613602, EPI2613606, EPI2613599, EPI2613605, EPI2613601, and EPI2613600; A/red-tailed hawk/New York/23–005536-001-original/2023: EPI2613635, EPI2613636, EPI2613634, EPI2613638, EPI2613631, EPI2613637, EPI2613633, and EPI2613632; A/American crow/New York/23–005128-001-original/2023: EPI2613611, EPI2613612, EPI2613610, EPI2613614, EPI2613607, EPI2613613, EPI2613609, and EPI2613608; A/peregrine falcon/New York/23–005700-001-original/2023: EPI2613699, EPI2613700, EPI2613698, EPI2613702, EPI2613695, EPI2613701, EPI2613697, and EPI2613696; A/Canada goose/New York/23–005698-001-original/2023: EPI2613683, EPI2613684, EPI2613682, EPI2613686, EPI2613679, EPI2613685, EPI2613681, and EPI2613680; A/Canada goose/New York/23–005699-001-original/2023: EPI2613691, EPI2613692, EPI2613690, EPI2613694, EPI2613687, EPI2613693, EPI2613689, and EPI2613688; A/American crow/New York/23–005695-001-original/2023: EPI2613675, EPI2613676, EPI2613674, EPI2613678, EPI2613671, EPI2613677, EPI2613673, and EPI2613672; A/Canada goose/New York/23–006363-001-original/2023: EPI2613779, EPI2613780, EPI2613778, EPI2613782, EPI2613775, EPI2613781, EPI2613777, and EPI2613776; A/American crow/New York/23–006663-001-original/2023: EPI2613819, EPI2613820, EPI2613818, EPI2613822, EPI2613815, EPI2613821, EPI2613817, and EPI2613816; A/Canada goose/New York/23–006664-001-original/2023: EPI2613827, EPI2613828, EPI2613826, EPI2613830, EPI2613823, EPI2613829, EPI2613825, and EPI2613824; A/Canada goose/New York/23–008112-001-original/2023: EPI2614003, EPI2614004, EPI2614002, EPI2614006, EPI2613999, EPI2614005, EPI2614001, and EPI2614000; A/American crow/New York/23–009037-001-original/2023: EPI2613979, EPI2613980, EPI2613978, EPI2613982, EPI2613975, EPI2613981, EPI2613977, and EPI2613976; A/peregrine falcon/New York/23–009036-001-original/2023: EPI2613971, EPI2613972, EPI2613970, EPI2613974, EPI2613967, EPI2613973, EPI2613969, and EPI2613968; A/American crow/New York/23–009035-001-original/2023: EPI2613963, EPI2613964, EPI2613962, EPI2613966, EPI2613959, EPI2613965, EPI2613961, and EPI2613960; A/red-tailed hawk/Connecticut/23–009886-001-original/2023: EPI2614123, EPI2614124, EPI2614122, EPI2614126, EPI2614119, EPI2614125, EPI2614121, and EPI2614120; A/red-tailed hawk/Connecticut/23–009880-001-original/2023: EPI2614115, EPI2614116, EPI2614114, EPI2614118, EPI2614111, EPI2614117, EPI2614113, and EPI2614112; A/turkey vulture/New York/23–011005-001-original/2023: EPI2614171, EPI2614172, EPI2614170, EPI2614174, EPI2614167, EPI2614173, EPI2614169, and EPI2614168; A/red-shouldered hawk/New York/23–012563-001-original/2023: EPI2614339, EPI2614340, EPI2614338, EPI2614342, EPI2614335, EPI2614341, EPI2614337, and EPI2614336; A/Canada goose/New York/23–013244-001-original/2023: EPI2614395, EPI2614396, EPI2614394, EPI2614398, EPI2614391, EPI2614397, EPI2614393, and EPI2614392; A/American crow/New York/23–014010-001-original/2023: EPI2614427, EPI2614428, EPI2614426, EPI2614430, EPI2614423, EPI2614429, EPI2614425, and EPI2614424; A/Canada goose/New York/23–014011-001-original/2023: EPI2614435, EPI2614436, EPI2614434, EPI2614438, EPI2614431, EPI2614437, EPI2614433, and EPI2614432.

## Data Availability

Sequences have been uploaded to GenBank and can be retrieved under the following identifiers: OR818561, OR818562, OR818563, OR818564, OR 818565, OR818566, OR818567, and OR818568 for (A/Red tailed hawk/New York//NYCVH-22-8477/8477/2022(H5N1)); OR818637, OR818638, OR818639, OR818640, OR818641, OR818642, OR818643 and OR818644 for (A/Canada goose/New York/NYCVH-22-6038/2022(H5N1)); OR818684, OR818685, OR818686, OR818687, OR818688, OR818689, OR818690 and OR818691 for (A/Canada goose/New York/NYCVH-22-9190/2022(H5N1)); OR819057, OR819058, OR819059, OR819060, OR819061, OR819062, OR819063 and OR819064 for (A/peregrine falcon/New York/NYCVH-160820/2022(H5N1)); OR858836, OR858837, OR858838, OR858839, OR858840, OR858841, OR858842 and OR858843 for (A/Canada goose/New York/NYCVH-23-453/2023(H5N1)); OR819337, OR819338, OR819339, OR819340, OR819341, OR819342, OR819343 and OR819344 for (A/chicken/New York/NYCVH-168127/2023(H5N1)).

## SUPPLEMENTAL MATERIAL

Figure S1 jvi.00626-24-s0001.tif HPAI H5N1 detections in New York, New Jersey, and Connecticut August 2022-April 2023.

ASM does not own the copyrights to Supplemental Material that may be linked to, or accessed through, an article. The authors have granted ASM a non-exclusive, world-wide license to publish the Supplemental Material files. Please contact the corresponding author directly for reuse.
