# Supplementary figures and images for: Detection of clade 2.3.4.4b highly pathogenic H5N1 influenza virus in New York City
Source: J Virol. 2024 May 15;98(6):e00626-24. doi: 10.1128/jvi.00626-24 (PMC11237497; doi:10.1128/jvi.00626-24)

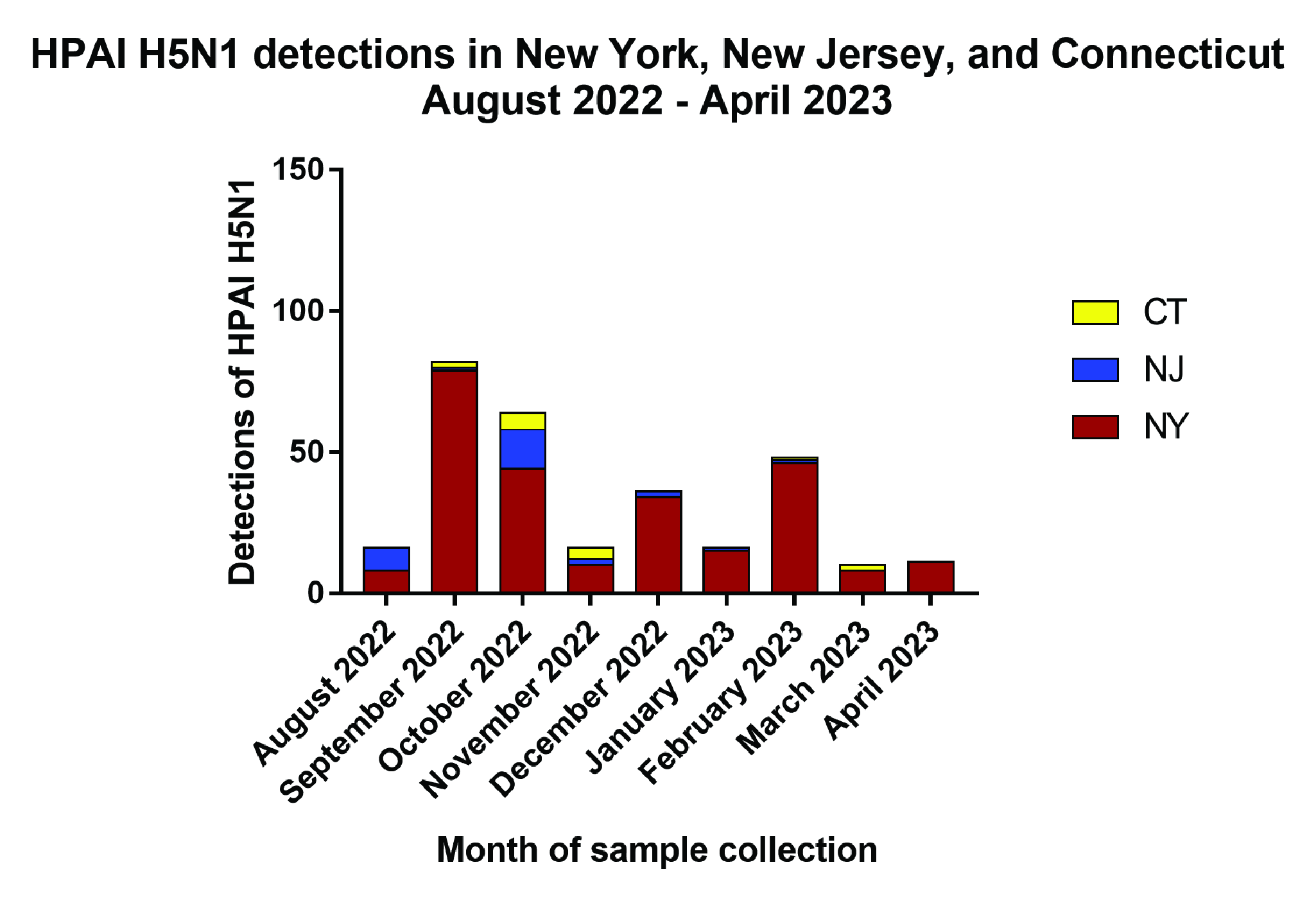

Supplement: Figure S1 — HPAI H5N1 detections in New York, New Jersey, and Connecticut August 2022-April 2023. [file jvi.00626-24-s0001.tif]
